# Physiological Characterization of *Sulfolobus acidocaldarius* in a Controlled Bioreactor Environment

**DOI:** 10.3390/ijerph18115532

**Published:** 2021-05-21

**Authors:** Kerstin Rastädter, David Johannes Wurm, Oliver Spadiut, Julian Quehenberger

**Affiliations:** Research Division Biochemical Engineering, Faculty of Technical Chemistry, Institute of Chemical, Environmental and Bioscience Engineering, TU Wien, 1060 Vienna, Austria; kerstin.rastaedter@tuwien.ac.at (K.R.); david.wurm@tuwien.ac.at (D.J.W.); oliver.spadiut@tuwien.ac.at (O.S.)

**Keywords:** *Sulfolobus acidocaldarius*, characterization, chemostat, dilution rate, substrate affinity, trehalose

## Abstract

The crenarchaeal model organism *Sulfolobus acidocaldarius* is typically cultivated in shake flasks. Although shake flasks represent the state-of-the-art for the cultivation of this microorganism, in these systems crucial process parameters, like pH or substrate availability, are only set initially, but cannot be controlled during the cultivation process. As a result, a thorough characterization of growth parameters under controlled conditions is still missing for *S. acidocaldarius*. In this study, we conducted chemostat cultivations at 75 °C using a growth medium containing L-glutamate and D-glucose as main carbon sources. Different pH values and dilution rates were applied with the goal to physiologically characterize the organism in a controlled bioreactor environment. Under these controlled conditions a pH optimum of 3.0 was determined. Washout of the cells occurred at a dilution rate of 0.097 h^−1^ and the optimal productivity of biomass was observed at a dilution rate of 0.062 h^−1^. While both carbon sources were taken up by *S. acidocaldarius* concomitantly, a 6.6-fold higher affinity for L-glutamate was shown. When exposed to suboptimal growth conditions, *S. acidocaldarius* reacted with a change in the respiratory behavior and an increased trehalose production rate in addition to a decreased growth rate.

## 1. Introduction

The extremophile *Sulfolobus acidocaldarius* is a widely used model organism of the phylum Crenarchaeota. Its genome sequence is available [1] and a powerful genetic tool box for investigating and engineering gene functions has been developed [2] and is continuously being improved [3]. The organism thrives in a temperature range of 65 to 85 °C and at a pH ranging from pH 2.0 to 5.5 [4], while under laboratory conditions it is generally cultivated at temperatures of 70 to 75 °C and at a pH of 2.25 to 3.5 [5,6,7,8,9,10]. Most research with this organism is performed in batch cultivations using shake flasks as culture vessels [5,7,11]. However, a continuous cultivation in a stirred-tank reactor (CSTR) with the possibility to monitor and control pH, temperature and dissolved oxygen content (dO_2_) is the basis for stable growth conditions, thereby allowing the acquisition of reliable and reproducible process data [12]. Furthermore, providing the substrate in limiting amounts might also represent conditions that resembles the natural habitat of most microorganism more closely than the nutrient enriched system of batch cultivations [13,14,15]. In chemostat cultures the growth rate, biomass, substrate and product concentration remain constant after reaching steady state [16], thus making it a reliable tool for strain characterization as shown for a variety of microorganisms [17,18,19]. Critical parameters for bioprocess development, such as the optimal and critical dilution rate, affinity constants for substrates as well as maintenance coefficient, can be readily determined in a CSTR. All these parameters are missing in the current literature of Sulfolobales.

In a CSTR a potent technique to assess the physiological condition of an organism is the monitoring of the respiratory behavior, expressed as the respiratory quotient (RQ). The RQ is the molar ratio of produced CO_2_ to consumed O_2_ and depends on the state of oxidation of the metabolized substrate and the utilized catabolic pathways [20]. Higher RQs than the theoretical value for complete oxidation of the respective substrates imply incomplete substrate utilization and potential waste of energy, while lower RQs can signify a change in the carbon flow towards metabolites and hint towards a less efficient utilization of O_2_. A further way to monitor metabolic stress is the direct measurement of metabolites in the culture supernatant. *S. acidocaldarius* produces the non-reducing disaccharide trehalose, a compound that can be found in all three domains of life, where it functions as a general protecting agent against unfavorable environmental conditions [21].

In this study, the strain *S. acidocaldarius* DSM639 was grown in continuous cultivation on a medium containing L-glutamate and D-glucose as main carbon sources and was physiologically characterized regarding its pH optimum, maximum growth rate, specific substrate uptake rates and its stress response indicated by trehalose production and respiratory behavior.

## 2. Materials and Methods

### 2.1. Bioreactor Cultivations

*S. acidocaldarius* DSM639, obtained at German Collection of Microorganisms and Cell Cultures (DSMZ, Braunschweig, Germany), was continuously cultivated in a DASbox Mini Bioreactor System (Eppendorf AG, Hamburg, Germany) with a working volume of 200 mL per vessel. Four parallel bioreactors, filled with Vienna Defined (VD) Medium [22] with modified carbon source concentrations (2 g/L monosodium glutamate (MSG), 1 g/L D-glucose) were inoculated with a starting OD_600_ of 0.17, yielding a total batch volume of 150 mL. Feed used during fed-batch and chemostat phases contained a 5-times concentrated VD Medium with modified carbon source concentrations (9.5 g/L MSG, 4.5 g/L D-glucose and a minute amount of 0.5 g/L NZ-amine to rule out any long-term nutrient deficiencies). Fed-batch phase was performed with a continuous feed rate of 3.0 mL/h until a reactor volume of 200 mL was reached. During the following chemostat phase, the feed rate was set to 6.0 mL/h (*D* = 0.03 h^−1^). To maintain a constant volume of 200 mL, broth was withdrawn from the reactor via a bleed tube at a fixed height. The reactors were stirred with 600 rpm and supplied with 0.3 vvm (volumes gas flow per working volume per minute; equal to: 3.6 sL/h; liter at standardized conditions, 273.15 K and 1 bar per hour) pressurized air. A constant growth temperature of 75 °C was achieved by submerging the reactor vessels in a stirred heated oil bath. pH was measured with an EasyFerm Plus K8 120 electrode (Hamilton, OH, USA) and was adjusted by automatic addition of 4.8% H_2_SO_4_. Dissolved oxygen (dO_2_) was monitored by a VisiFerm DO225 probe (Hamilton, OH, USA). CO_2_ and O_2_ concentrations were measured using the DASGIP GA4 exhaust analyzer (Eppendorf AG, Hamburg, Germany).

To assess reproducibility three bioreactors of the DASbox Mini Bioreactor System were subjected to the same conditions (75 °C, pH 3.0, *D* = 0.03 h^−1^). Table 1 gives an overview of the experiments performed in this study.

Since the cultivation performed at pH 2.0 in the DASbox Mini Bioreactor System yielded an inexplicably high CO_2_ yield and consequently a C-balance that significantly exceeded a value of 1.0, this experiment was excluded from the study and was instead repeated in a 2 L Biostat A-plus bioreactor (Sartorius, Goettingen, Germany). The reactor was stirred at 300 rpm and supplied with 0.23 vvm (0.45 sL/h) pressurized air. The pH was measured with an EasyFerm Plus electrode (Hamilton, OH, USA) and controlled via automatic addition of 4.8% H_2_SO_4_. CO_2_ and O_2_ concentrations in the exhaust gas were measured using a gas analyzing unit (Müller Systems AG, Esslingen, Switzerland). The batch phase was started with 1.5 L and chemostat culturing was done at 2 L and a dilution rate of 0.03 h^−1^. During fed-batch an exponential feed was applied, starting with 14.8 g/h and a growth rate of 0.035 h^−1^. The cultivation was controlled using the Lucullus process control system (SecureCell AG, Urdorf, Switzerland). All other process parameters were set as mentioned for the DASbox Mini Bioreactor System.

### 2.2. Biomass Determination

Optical density, OD_600_, was determined photometrically at 600 nm with a spectrophotometer against a blank of deionized water (ONDA V-10 PLUS, XS instruments, Carpi, Italy). Samples were diluted with deionized water to stay in the linear range of the photometer. Due to the low biomass concentration and low reactor volume, dry cell weight (DCW) was not determined. A previously determined correlation factor of 0.586 g/L (see Supplementary Figure S1) between DCW concentration and OD_600_ was applied.

### 2.3. Substrate and Metabolites Analytics

At each sampling point, 1 mL of culture broth was centrifuged at 10,000 g for 10 min at 4 °C. The obtained supernatant was analyzed for its substrate and metabolite composition. D-glucose and trehalose concentrations in the supernatant were determined with an Aminex HPX-87H column (300 × 7.8 mm, Bio-Rad, Hercules, CA, USA) employing an Ultimate 3000 high-performance liquid chromatography (HPLC) system (Thermo Fisher Scientific, Waltham, MA, USA). 10 µL sample were analyzed at a flow rate of 0.6 mL/min and a column temperature of 60 °C. 4 mM H_2_SO_4_ served as mobile phase. For quantitative determination a RI detector (RefractoMax 520, Thermo Fisher Scientific, Waltham, MA, USA) and a UV detector (VH-D10-A, Thermo Fisher Scientific, Waltham, MA, USA) at 210 nm were used. Chromeleon 7.2.6 Chromatography Data System (Thermo Fisher Scientific, Waltham, MA, USA) was used for control and data analysis. Glutamic acid was determined via a photometric assay using a Cedex Bio HT Analyzer (Roche, Basel, Switzerland). The obtained concentration of glutamic acid was converted to the used substrate MSG by multiplication with the factor 1.15.

### 2.4. Calculation of Rates, Substrate Affinity Constants and Maintenance Coefficient

All rates, balances and yields were determined at each sampling point and then for each condition (pH & dilution rate) the mean value of the different sampling points was calculated.

*Dilution rate, D* [h^−1^], in the CSTR was calculated as the sum of feed and acid addition [L/h] divided by the reactor volume [L].

*Dwell time, τ* [h], is the invers value of the dilution rate.

*Specific growth rate,* µ [h^−1^], was determined as the difference in DCW between two sampling points divided by the average DCW between the sampling points per hour by taking into account the loss of biomass via the bleed.

(1) $$µ=\frac{\Delta X+\Delta V_{bleed}*\bar{x}}{\bar{X}*\Delta t}$$

Δ*t* [h]  time between two sampling points

Δ*X* [g]  difference of total DCW in broth between the two sampling points

Δ*V_bleed_* [L]  bleed volume removed between the two sampling points

$\bar{x}$ [g/L]  average DCW concentration between sampling points

$\bar{X}$ [g]  average biomass in broth between sampling points

Cell productivity, biomass space time yield, D*x [g L^−1^ h^−1^], was calculated as the product of DCW concentration [g/L] and *D* [h^−1^].

*Specific substrate uptake rates* for D-glucose, *q_Glc_* [g_Glc_ g(*X*)^−1^ h^−1^], and L-glutamate, *q_MSG_* [g_MSG_ g(*X*)^−1^ h^−1^] (shown as MSG per DCW per hour), were calculated for every time span between sampling points as follows:

(2) $$q_{S}=\frac{\Delta S_{reactor}+S_{in}-S_{out}}{\Delta t*\bar{X}\;}$$

Δ*S_reactor_* [g]  difference of amount of substrate in broth between the two sampling points

*S_in_* [g]  substrate supplied to bioreactor within the time period

*S_out_* [g]  substrate discharged via the bleed within the time period

*Specific production rate* of extracellular trehalose *q_Tre_* [g_Tre_ g(*X*)^−1^ h^−1^] was calculated according to:

(3) $$q_{P}=\frac{\Delta P_{reactor}+P_{in}-P_{out}}{\Delta t*\bar{X}\;}$$

Δ*P_reactor_* [g]  difference of amount of product in broth between the two sampling points

*P_in_* [g]  product supplied to bioreactor within the time period

*P_out_* [g]  product discharged via the bleed within the time period

*Biomass Yield, *Y*_*X*/*S*_* [g(*X*)/g(*S*)], was calculated as the quotient of µ [h^−1^] and q_*S*_ [g(*S*) g(*X*)^−1^ h^−1^].

*CO_2_ yield, Y_CO2/S_* [C-mol_CO2_/C-mol_S_] was calculated as the quotient of the specific CO_2_ evolution rate [C-mol_CO2_ g(*X*)^−1^ h^−1^] and q_*S*_ [C-mol_S_ g(*X*)^−1^ h^−1^].

*Trehalose yield, *Y*_*Tre*/*S*_* [C-mol_Tre_/C-mol_S_] was calculated as the quotient of q_Tre_ [C-mol_Tre_ g(*X*)^−1^ h^−1^] and q_*S*_ [C-mol_S_ g(*X*)^−1^ h^−1^].

*C-balance* was determined as the sum of *Y*_*X*/*S*_, *Y*_*CO2*/__*S*_, and *Y*_*Tre*/*S*_, all in carbon-mol per carbon-mol. A C-balance close to 1.0 implies that all carbon atoms provided via substrate can be accounted for and are recovered either in the biomass (*Y*_*X*/*S*_), in the exhaust gas (*Y*_*CO2*/*S*_) or in metabolites (*Y*_*Tre*/*S*_).

*Respiratory quotient, RQ,* was calculated as the quotient of the carbon evolution rate [mmol L^−1^ h^−1^] and the oxygen uptake rate [mmol L^−1^ h^−1^]. Oxygen uptake rate and CO_2_ evolution rate were calculated by measuring the effluent concentrations of oxygen and CO_2_.

*Specific carbon dioxide production rate, q_CO2_* [mmol g(*X*)^−1^ h^−1^], was determined by dividing the carbon dioxide production rate by the DCW concentration.

*Specific oxygen consumption rate, q_O2_* [mmol g(*X*)^−1^ h^−1^], was determined by dividing the oxygen uptake rate by the DCW concentration.

*Substrate affinity constants, Ks* [g/L] for D-glucose and MSG and the *maximum growth rate*, µ*_max_* [h^−1^], were fitted by using the program SigmaPlot 14 (Systat Software, San Jose, CA, USA). The growth rate was plotted versus the substrate concentration in the supernatant and a Monod function (Equation (4)) was fitted accordingly. The standard errors for K_S_ and µ*_max_* describing the quality of the fit were calculated with SigmaPlot.

(4) $$µ={µ}_{\max}\frac{S}{S+K_{S}}$$

µ [h^−1^]  growth rate calculated for each applied dilution rate

*S* [g/L]  substrate concentration, measured in the supernatant

With the obtained K_S_ values and µ*_max_* the *critical dilution rate, D_crit_* [h^−1^] for both D-glucose and MSG were determined according to:

(5) $$D_{crit}={µ}_{max}\frac{S_{0}}{K_{s}+S_{0}}$$

µ**_max_** [h^−1^]  maximum growth rate determined via Equation (4)

*S*_0_ [g/L]  substrate concentration (D-glucose or MSG) in the supplied feed

*Optimal dilution rate*, *D_opt_* [h^−1^], for maximum cell productivity, was calculated as follows:

(6) $$D_{opt}={µ}_{max}\left(1-\sqrt{\frac{K_{s}}{K_{s}+S_{0}}}\;\right)$$

To determine the specific *maintenance coefficient, m_s_* [g(*S*) g(*X*)^−1^ h^−1^], Equation (7) was rearranged to Equation (8). Subsequently, the overall specific substrate uptake rate q_*S*_ (q_Glc_ + q_MSG_) was plotted against the specific growth rates resulting in a line with a slope equivalent to 1/*Y*_*X*/S_^*^ and an offset equivalent to m_s_.

(7) $$µ=\left(q_{S}-m_{S}\right)*Y_{X/S}^{*}$$

(8) $$q_{S}=\frac{1}{Y_{X/S}^{*}}*µ+m_{S}$$

q_*S*_ [g(*S*) g(*X*)^−1^ h^−^]  specific substrate uptake rate, the sum of q_Glc_ and q_MSG_

*Y*_*X*/S_^*^ [g/g]  true biomass yield, used solely for growth

*Standard deviation, STD* [%], for each parameter of the reproducibility experiments was calculated as the square root of the variance divided by its mean.

## 3. Results

Reproducibility experiments, in which three reactors were subjected to the same conditions (75 °C, pH 3.0, *D* = 0.03 h^−1^), showed a standard deviation (STD) <3% for OD, µ and q_MSG_, whereas q_Glc_ and q_Tre_ exhibited a STD of <10%. In the following experiments, each condition (shift of pH or dilution rate) was maintained for at least four theoretical dwell times. This number of dwell times, required for reaching steady state, was determined during the reproducibility studies (data not shown).

### 3.1. Effect of pH on Strain Physiology

Since the pH optimum of this organism is still disputed in literature and recently Cobban et al. [11] showed data conflicting with the broad consensus of the optimal pH of 3 to 3.5 [5,6,7,8,9,10], we determined the pH optimum by shifting the pH from 2.0 to 4.0 in increments of 0.5 pH units at a constant growth temperature of 75 °C and dilution rate of 0.03 h^−1^.

**Figure 1:**
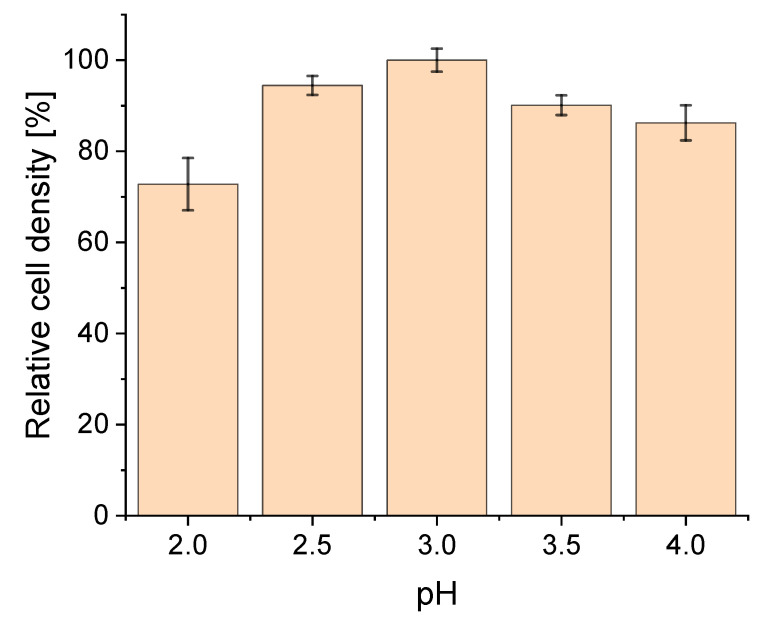
Relative cell density [%] of *Sulfolobus acidocaldarius* in response to different pH values (2.0–4.0) in a chemostat cultivation at 75 °C and a set dilution rate of 0.03 h^−1^. Error bars indicate the standard deviation of cell density between sampling points after reaching steady state.

In our studies, highest cell densities were reached at pH 3.0 (Figure 1). Upon reducing the pH to 2.5 and 2.0, the cell density decreased by 9% and 27%, respectively. Increasing the pH to 3.5 and 4.0 resulted in a reduction of cell density by 13% and 14%, respectively.

**Figure 2:**
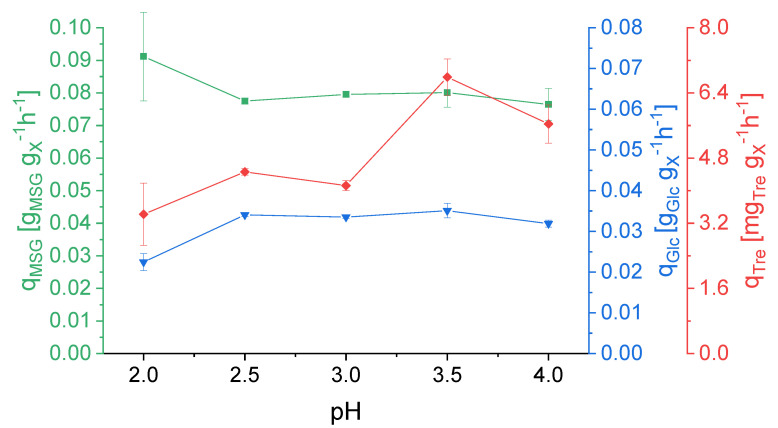
Specific uptake rates for L-glutamate (q_MSG_) and D-glucose (q_Glc_) and specific production rate of trehalose (q_Tre_) in dependence of the pH value. Error bars indicate the difference between the various sampling points after reaching steady state in the continuous cultivation.

The specific glutamate uptake rate, q_MSG_, was constant throughout the pH changes with only a slight, nonsignificant increase at pH 2.0 (Figure 2). The specific glucose uptake rate, q_Glc_, decreased considerably at pH 2.0. The specific production rate of trehalose, q_Tre_, increased when the culture was subjected to pH values of 3.5 and 4.0 (1.64- and 1.37-fold, respectively), implicating a metabolic shift at higher pH values.

**Figure 3:**
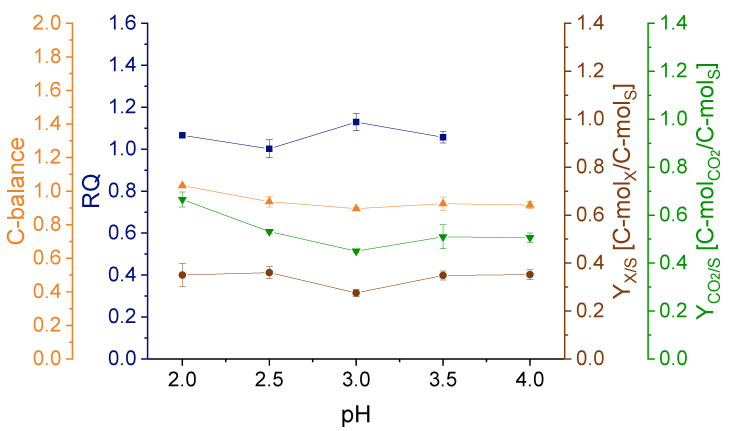
C-balance, respiratory quotient (RQ), biomass yield (*Y*_*X*/*S*_) and CO_2_ yield (*Y*_*CO2*/__*S*_) in dependence of the pH value. Error bars indicate the difference between the various sampling points after reaching steady state in the continuous cultivation. The RQ at pH 4.0 could not be calculated due to a malfunction of the O_2_ analyzer during the cultivation.

The C-balance of all cultivations closed at 1.0 ± 0.1. Also, RQ and biomass yield remained constant throughout all investigated pH values (Figure 3). Solely the CO_2_ yield increased at pH 2.0 compared to the other pH conditions.

### 3.2. Effect of Dilution Rate on Strain Physiology

**Figure 4:**
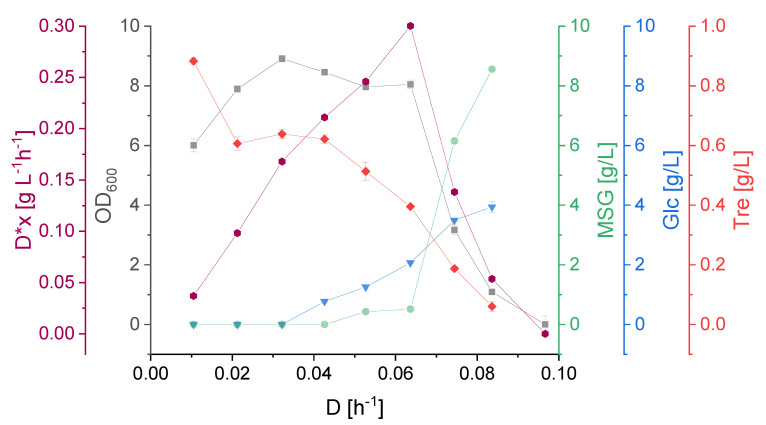
D-x graph: space time yield (D*x) and cell density (OD_600_), monosodium glutamate (MSG), D-glucose concentration (Glc) and trehalose (Tre) concentration in the supernatant as a function of the applied dilution rate (D). Error bars indicate the difference between the various sampling points after reaching steady state in the continuous cultivation.

For determination of µ*_max_*, the optimum and critical dilution rate, the substrate affinity constants as well as the maintenance coefficient, *S. acidocaldarius* cultures were subjected to different dilution rates ranging from 0.010 to 0.097 h^−1^ at a constant pH of 3.0. The dilution rate was stepwise increased by increments of ~0.011 h^−1^. D-glucose started to accumulate prior to L-glutamate while the extracellular trehalose concentration decreased as a result of increasing dilution rates (Figure 4). The cell density was the highest at a dilution rate of 0.032 h^−1^. However, the biomass space time yield reached its maximum at *D* = 0.062 h^−1^ with a value of 0.3 g L^−1^ h^−1^. A dilution rate of 0.074 h^−1^ led to a rapid decrease of biomass concentration in the reactor. Washout, defined as the dilution rate at which no steady state could be established, occurred between 0.084 and 0.097 h^−1^.

**Figure 5:**
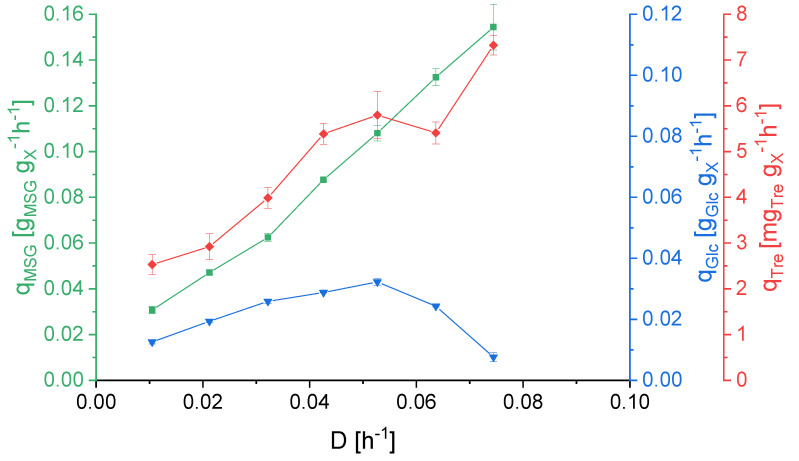
Specific substrate uptake rates for L-glutamate (q_MSG_) and for D-glucose (q_Glc_) and specific production rates of trehalose (q_Tre_) in dependence of the different applied dilution rates. The specific rates at the dilution rate of 0.084 h^−1^ are not depicted as this dilution rate yielded highly variable rates due to it lying too close to the point of washout. Error bars indicate the difference between the various sampling points after reaching steady state in the continuous cultivation.

Figure 5 shows the difference between the uptake rates of D-glucose and L-glutamate. While q_MSG_ increased with the applied dilution rate, q_Glc_ reached its peak at 0.053 h^−1^. Then q_Glc_ decreased, indicating that the metabolic capacity had been exhausted for this substrate. The specific trehalose production was increasing in response to increasing dilution rates. This is a further indication for the role of trehalose production as part of a stress response [23]. Rising dilution rates put the cell under stress due to the growth rate approaching µ*_max_* and the organism is gradually approaching its maximal metabolic capacity [24,25].

**Figure 6:**
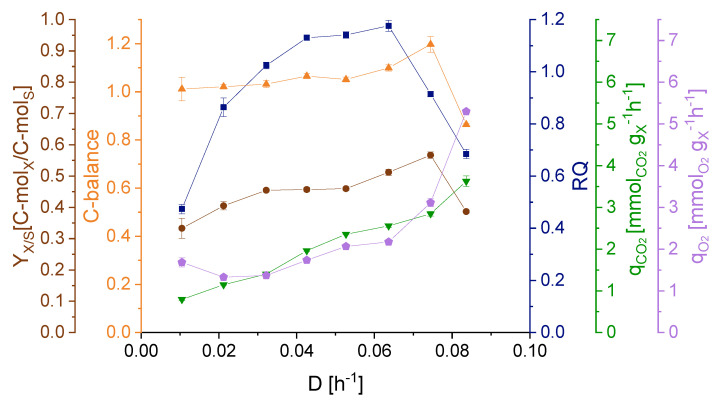
Biomass yield (*Y*_*X*/*S*_), C-balance, respiratory quotient (RQ), specific carbon dioxide production rate (q_CO2_) and specific oxygen consumption rate (q_O2_) at each dilution rate. Error bars indicate the difference between the various sampling points after reaching steady state in the continuous cultivation.

The course of the RQ values mirrored the q_Glc_ trend in relation to the different applied dilution rates (Figure 6). The highest RQ of 1.18 was determined at *D* = 0.064 h^−1^ and the lowest at *D* = 0.010 h^−1^. These decreased RQ values at the lower and higher dilution rates, indicate a lower CO_2_ production in relation to the O_2_ consumption. Since q_O2_ varies more greatly with the dilution rates than q_CO2_, the main reason for the shifted RQ values is a change in the specific oxygen uptake rate, q_O2_. A less efficient oxygen utilization in the respiratory chain as a result of the dilution rate is a possible explanation for the increasing demand for oxygen. The C-balance was around 1.0 until a dilution rate of 0.074 h^−1^. The over- and undershooting of the C-balance at dilution rates of 0.074 h^−1^ and higher can be interpreted as a sign for the culture reaching a non-steady state and approaching the point of washout.

### 3.3. K_S_ Values, Optimal and Critical Dilution Rate

Based on the Monod fit of µ plotted versus the substrate concentration, the affinity constants for the used substrates, L-glutamate and D-glucose, were calculated. As shown in Figure 4, D-glucose started to accumulate at a dilution rate of 0.042 h^−1^ while L-glutamate did not accumulate until a dilution rate of 0.052 h^−1^. This is also reflected in the calculated K_S_ values (Table 2). According to these, the affinity of *S. acidocaldarius* for D-glucose (0.99 g/L) is 6.6 times lower than for MSG (0.15 g/L). The resulting theoretical µ*_max_*, determined via the Monod fit, also differed for the two substrates, 0.077 h^−1^ for MSG and 0.095 h^−1^ for D-glucose. Based on the exponential increase of the CO_2_ concentration in the exhaust gas during the batch phase at the beginning of the cultivation, µ*_max_* was determined as 0.097 h^−1^.

The optimal dilution rate calculated in this study is different in depending on the different substrate uptakes (Table 2). A lower D_opt_ of 0.055 h^−1^ is postulated based on the utilization of D-glucose. However, for the main substrate L-glutamate an optimal dilution rate of 0.068 h^−1^ is calculated, which is supported by the D*x diagram (Figure 4). The considerable difference in K_S_ values for the substrates causes the divergence of the actual D_opt_ to the one presumed based on D-glucose, while the uptake of L-glutamate increased the optimal dilution rate. The calculated D_crit_ of around 0.077 h^−1^ after which washout should occur is slightly lower compared to the obtained experimental data (*D* = 0.084 h^−1^, Figure 4).

### 3.4. Maintenance

Maintenance respiration in thermoacidophiles is assumed to be relatively high [26], due to the need for retaining a near neutral cytosolic pH of 6.5 [27] as well as for repairing DNA and protein damage as a result of the high temperature [28] thereby possibly draining the supply of carbon sources available for biomass growth. Therefore, to further characterize *S. acidocaldarius*, its maintenance coefficient was calculated by plotting q_*S*_ (q_Glc_ + q_MSG_) versus growth rate. The maintenance coefficient for the total substrate was determined according to Equation (8), as well as the true biomass yield (the biomass yield per substrate used solely for growth). For this calculation, just the values of the dilution rates between 0.010 and 0.064 h^−1^ were considered, since higher dilution rate showed a flattening of the curve as the q_*S*_ values differed only marginally. Thereby, a true biomass yield of 0.42 g(*X*)/g(*S*) was determined. The calculated maintenance requirement, m_S_, of 0.017 g(*S*) g(*X*)^−1^ h^−1^ for *S. acidocaldarius* for the mixed substrate feed with D-glucose and L-glutamate is surprisingly low, since Korz et al. postulated the maintenance coefficient in *Escherichia coli* to be 0.025 gS/gX/h cultivated in D-glucose or glycerol [29]. Nevertheless, in relative terms the ratio of m_S_ to the observed total q_*S*_ is around 10% at a dilution rate of 0.075 h^−1^ and increases with decreasing dilution rates to 39% at 0.010 h^−1^. Even though similar values of maintenance coefficients were observed in other organisms and the biomass yield for D-glucose is also comparable to *E. coli* [30], the possible q_*S*_ values in bacteria is higher by up to 3.42–12.8 fold [30]. Hence, in that perspective the ratio maintenance coefficient to substrate uptake rates is much higher in this extremophilic organism. Studies in other bacteria showed a high variability in maintenance coefficients within the same species. This variation, in addition to differently specified coefficient units, impedes comparison with other species. Nevertheless, a trend towards higher maintenance coefficients was observed under anaerobic cultivation conditions [31,32]. Unfortunately, no comparative results for maintenance coefficient in Archaea was found.

## 4. Discussion

While there is broad consensus on the optimal growth temperature of *S. acidocaldarius* being at 75 °C [7,8,9,10,33] the described optimal cultivation pH still varies [5,6,7,8,9,10]. Our study determined a pH optimum of 3.0 in regards to the cell density during continuous cultivation (Figure 2), contradicting the recent publication by Cobban et al. [11], in which higher biomass yields were observed at pH 2.0 and 4.0 compared to pH 3.0. A possible reason for the differences could be the used cultivation temperature for their experiments of 70 °C and that cultivations were performed in batch mode using a different medium.

The applied pH influenced the specific glucose uptake rate and the specific trehalose production rate (Figure 3). According to the obtained data, higher pH triggered trehalose production while lower pH had no effect. Koerdt et al. [34] showed increasing biofilm formation with increasing pHs as well as upregulated sugar transport for the formation of the biofilm. In the present study, specific trehalose production was upregulated at higher pHs, possibly going hand-in-hand with the observed increased biofilm formation at higher pH values by Koerdt et al.

The substrate uptake rates (Figure 5) are in accordance with the obtained K_s_ values (Table 2). D-glucose, like other saccharides, is transported into the cell via an ATP binding cassettes (ABC) transporter [35,36]. The transporter for L-glutamate has not been identified yet. Until now, ABC transporters were only found to be responsible for di- and oligopeptide uptake in *Saccharolobus solfataricus* (*Sulfolobus solfataricus*) and *Sulfurisphaera tokodaii* (*Sulfolobus tokodaii*) [35]. However, these are all close relatives of *S. acidocaldarius*. Amino acids have also been shown to be transported into organisms by facilitated diffusion [37]. Despite the organism’s preference of MSG over D-glucose, the presence of the latter is still beneficial. The specific uptake rate increases until *D* = 0.053 h^−1^, confirming the organism’s capacity for metabolizing D-glucose and MSG concomitantly.

Upon changing dilution rates, the respiratory behavior, as shown by the RQ values, as well as the calculated C-balances (Figure 6) indicate a shift in the carbon flow. Additionally, the elevated oxygen consumption at low and high dilution rates further propose an impairment in the respiratory chain, implying a higher O_2_ need for substrate metabolization.

With the obtained data a range of 0.077 to 0.097 h^−1^ for µ*_max_* was identified. According to the exponential increase in the batch phase µ*_max_* is 0.097 h^−1^. In the applied chemostat cultivation µ*_max_* lies between 0.084 and 0.097 h^−1^. Based on the Monod fit for each substrate, µ*_max_* varies significantly between 0.077 h^−1^ for MSG and 0.095 h^−1^ for D-glucose. The overall range of obtained µ*_max_* is in accordance with data obtained in shake flask cultivation within the genus *Sulfolobus* spp. [22,38].

The characterization of *S. acidocaldarius* in a continuous bioreactor environment and determination of the relationship between pH, substrate uptake and trehalose production rate showed indications for distinctive stress responses at extreme pH values and dilution rates expressed as low RQ values, increased trehalose production and decreased specific glucose uptake rates. This study demonstrates the value of CSTR setups for fast and reliable generation of physiological parameters in a highly reproducible environment. As an outlook, the methodology described in this work can be used for medium optimization based on substrate affinity, control of biomass and metabolite production and optimization of time-space-yields in production processes in industrial settings, as well as the determination of operation windows of process conditions where robust cultivation of *S. acidocaldarius* is possible. Overall, with this study we could bring novel insight in the physiology of *S. acidocaldarius* under steady-state conditions and provide a basis for further bioprocess development, monitoring and control.

## 5. Conclusions

In this study, physiological characterization in a controlled chemostat cultivation using a modified VD Medium at a constant temperature of 75 °C was conducted. The pH optimum was confirmed to be 3.0. The affinity constants of the used carbon sources, D-glucose and MSG was determined. The specific glucose uptake rates in response to the dilution rates imply a possible overflow metabolism in which the substrate is incompletely oxidized despite the availability of oxygen which however has to be further investigated for possible verification. The maximum growth rate was determined to be 0.097 h^−1^. Respiratory behavior and trehalose production changed as a response to the different pH and dilution rate, indicating a stress response of the organism. All in all, it was shown that controlled cultivation conditions employing *S. acidocaldarius* are possible and beneficiary for further research topics of this crenarchaeal model organism and represent a solid foundation for further bioprocess development.

## Figures and Tables

**Table 1:** Experimental plan of this study: chemostat cultivations with a dilution rate (*D*) of 0.03 h^−1^ were performed at 5 different pH values. The following set of experiments where the dilution rate was varied from 0.010 h^−1^ to 0.097 h^−1^ was then performed at the prior determined pH optimum of 3.0.

Fixed Parameter		Variable Parameter	
*D*	0.03 h^−1^	pH	2.0, 2.5, 3.0, 3.5, 4.0
pH	3.0	*D*	0.010 h^−1^_,_ 0.021 h^−1^_,_ 0.032 h^−1^_,_ 0.043 h^−1^_,_ 0.053 h^−1^_,_ 0.064 h^−1^_,_ 0.074 h^−1^_,_ 0.084 h^−1^_,_ 0.097 h^−1^

**Table 2:** Affinity constants (K_S_) and maximum growth rates (µ*_max_*) for D-glucose and monosodium glutamate, obtained by fitting the growth rates versus the substrates concentration in the supernatant to the Monod equation with SigmaPlot. With the obtained values, the optimal (D_opt_) and critical dilution rate (D_crit_) were calculated.

Parameters	D-Glucose	Monosodium Glutamate (MSG)
µ*_max_* [h^−1^]	0.095 ± 0.004	0.077 ± 0.003
K_S_ [g/L]	0.99 ± 0.13	0.15 ± 0.04
D_opt_ [h^−1^]	0.055	0.068
D_crit_ [h^−1^]	0.078	0.077

## Supplementary Materials

The following are available online at https://www.mdpi.com/article/10.3390/ijerph18115532/s1, Figure S1: Correlation between dry cell weight (DCW) and OD_600_ of *Sulfolobus acidocaldarius.*
