# Supplementary material for: Physiological Characterization of Sulfolobus acidocaldarius in a Controlled Bioreactor Environment
Source: Int J Environ Res Public Health. 2021 May 21;18(11):5532. doi: 10.3390/ijerph18115532 (PMC8196767; doi:10.3390/ijerph18115532)
Supplement: Supplementary file 1 [file ijerph-18-05532-s001.zip › ijerph-1207682-supplementary.pdf]

Supplementary Materials

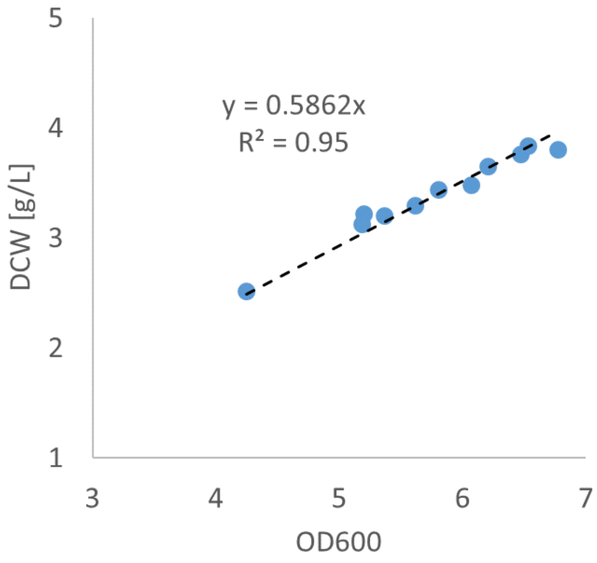

Figure S1. Correlation between dry cell weight (DCW) and OD_{600} of *Sulfolobus acidocaldarius*. DCW and OD_{600} values were obtained in a continuous cultivation in a bioreactor during a 240 hours timespan. The slope (0.586 g/L) was used as a conversion factor from OD_{600} to DCW.
